# Economic Evaluation of Free Prevention of Mother-to-Child Transmissions (PMTCT) Services to Non-South African Women Living in South Africa

**DOI:** 10.1177/11786329251316660

**Published:** 2025-02-13

**Authors:** Micheal Kofi Boachie, Vinayak Bhardwaj, Bontle Mamabolo, Winfrida Mdewa, Susan Goldstein, Karen Hofman, Evelyn Thsehla

**Affiliations:** SAMRC/Wits Centre for Health Economics and Decision Science – PRICELESS SA, School of Public Health, Faculty of Health Sciences, Wits University, Johannesburg, South Africa

**Keywords:** Migrants, HIV/AIDS, South Africa, PMTCT, cost-effectiveness

## Abstract

Approximately 1.33 million pregnancies are recorded in South Africa annually. About 30% of all pregnant women are HIV positive, posing a serious risk to unborn children. However, effective interventions such as prevention of mother-to-child transmissions (PMTCT) services have been shown to significantly reduce the risk of mother-to-child or vertical transmission. Migrant women in South Africa face challenges in accessing [free] healthcare services. This study aims to assess the cost-effectiveness of providing free PMTCT services to migrant women living in South Africa. We employed cost-effectiveness analysis methodology to establish the cost and outcomes (averted pediatric infections and averted disability-adjusted life years (DALYs)) associated with free PMTCT services for migrant women. The comparator was provision of only antenatal care (ANC) while the intervention was ANC + PMTCT services. A Microsoft Excel-based decision tree model was designed to achieve the study objectives. Data on costs and health outcomes for each intervention was sourced from the literature on HIV/AIDS. The prevalence-based study is conducted from a public sector healthcare payer perspective. Provision of ANC + PMTCT services to migrants will prevent 14 562 new infections among 52 762 HIV positive pregnant women. The estimated total expected cost of ANC + PMTCT service was US$52 889 per 1000 live births compared to US$191 000 for ANC only per 1000 live births. The expected cost for the do-nothing scenario was US$73 535 per 1000 live births. The expected health benefit (ie, averted DALYs) associated with do-nothing scenario, ANC, and ANC + PMTCT were 277, 265 and 76 DALYs, respectively. ANC + PMTCT service provision produces the lowest DALYs at lower cost thereby producing cost-saving of US$733/DALY averted per 1000 live births. Further, an average of US$1.5 million would be required annually to achieve 100% coverage of HIV+ migrant women. Therefore, provision of ANC and PMTCT services to migrant women is cost-effective when compared to not offering PMTCT services and allows the government to avoid the long-term cost of antiretroviral therapy (ART) provision.

## Introduction

South Africa (SA) is host to one of the largest migrant populations in Africa, with over 2.9 million immigrants, representing 5% of the population. Many migrants are from the Sub-Saharan African region, which is disproportionately burdened by HIV/AIDS.^1^ Immigrant female demographics also show that a large proportion are of childbearing age, who are likely to give birth within the destination country.^2^ This group by virtue of being female and migrant is vulnerable and at a higher risk of being infected with HIV.^3^ It is generally known that HIV infection in pregnancy is associated with increased maternal, fetal, and neonatal complications which if left untreated will lead to maternal and child mortality in-utero, at-birth or postnatally,^4^ indicating a need for [health] interventions that prevent mother-to-child transmission. Without intervention, vertical transmission of HIV from an HIV positive mother to her child during pregnancy, labor, delivery, or breastfeeding ranges from 15% to 45%.^5^ Recently, 232 newborns in the City of Tshwane Health District (Gauteng Province) tested positive for HIV and the poor uptake of vertical transmission interventions might be the reason for it.^6^

As people move across borders, the demand for healthcare services also increases in the host countries. Aside from the utility that migrants enjoy from good health, they also need to always be in a healthy state to benefit from the economic opportunities that their host countries present. Primary healthcare (PHC) services are available to all who live within South Africa’s borders, according to the Constitution, the National Health Act, and various legislation of the state.^7^ Therefore, healthcare workers provide PHC and emergency healthcare services to both citizens and non-citizens. They are, however, obligated by law to report undocumented migrants to assist the government in curbing illegal migration.^8^ Specifically, staff at healthcare facilities must find out the legal status of patients before providing care (except in an emergency).^9^ In some cases, provincial laws and regulations on health may be at odds with the national law to the extent that some patients may be denied access to care because they have foreign identity documents, for example, an asylum seeker permit, passport, or tourist/work visa, and these women often wait for hours without being attended to and are frequently turned away without receiving medical attention,^10^ with access to HIV services for migrants even more difficult during COVID-19 pandemic due to mistreatment and medical xenophobia.^11^ In 2023, the High Court in Johannesburg declared it unlawful not to provide free services to pregnant and lactating women and children under age 6.^12^ Consequently, South Africa has extended free maternal and child health services to all pregnant and lactating mothers and children below age 6, regardless of national documentation status. In practice this is not always the case, and women may not be treated because of discrimination, or lack of documentation. The antenatal care (ANC) program is now universally accessible and is offered as part of maternal and child health services which includes the Vertical Transmission Prevention program, formally known as prevention of mother-to-child transmission (PMTCT) program^13^—when referring to the program, the former.

The South African National Department of Health recommends a minimum of 5 ANC visits, which ideally begins at 20 weeks of gestation.^14,15^ In line with Basic Antenatal Care (BANC) Plus handbook and PMTCT program guidelines, the first ANC visit requires an HIV serology test and counseling, subsequently followed by a rapid HIV testing at each ANC visit together with counseling. Due to high prevalence and the adverse effects associated with infection, HIV screening and counseling during pregnancy is considered the most important screening investigation. Different from many other countries with a low HIV prevalence, South Africa does not consider HIV positive pregnant women as high-risk, thus they may continue receiving ANC at PHC level, granted no additional complications exist.^14^ Furthermore, HIV positive pregnant and/or breastfeeding women, and postnatal women within 1 year are eligible for lifelong antiretroviral therapy (ART), regardless of immunity status (CD4 count) or feeding choice.^15^ The implementation of the PMTCT program, since 2006, has reduced vertical transmission rate of HIV from 23% in 2003 to less than 1% in 2019.^16^ As of July 2024, South Africa has achieved an overall 97% coverage of lifelong ART among pregnant and breastfeeding women living with HIV, which is a positive step in preventing vertical transmissions.^17^

The non-uniform application of SA laws contributes to the differences in experience of PHC services or lack thereof by migrants, more especially those that are undocumented. This contributes to increased risk of vertical transmission during pregnancy, at birth and postnatally for this group. As noted by Bisnauth et al,^18^ an increasing number of foreign nationals access maternal, newborn and child health services at specific health facilities. A recent study at a different hospital has, however, shown no difference in the number of ANC visits, HIV prevalence and PMTCT treatment received between South African citizens and immigrants attending public healthcare facilities, but that migrants were more vulnerable due to poorer living conditions and lower incomes, and the fact that 10% of babies of migrants were born outside a medical facility.^19^ Other studies show that children born to migrants have increased risk of death relative to children of South African mothers.^20^

While legislation guarantees access to care for foreigners, barriers to access remain, with reports of some (undocumented) migrants seeking medicines from their home countries instead of accessing such services from within SA; language barriers and medical xenophobia are also barriers to accessing the free care.^11,21,22^ Free access to health services (ie, maternal and child health services) to foreigners is increasingly the subject of debate and controversy, and has been of concern for many due to medical xenophobia being practiced by some healthcare providers.^22-26^ At the same time, data on the cost and effectiveness of free PMTCT services for migrant women is scant. Evidence from other contexts shows that it is not cost saving to restrict access to healthcare to migrants,^27^ and that the cost of exclusion can be substantial,^28,29^ especially in the case of HIV.

The aim of this study is to establish the cost-effectiveness of providing free PMTCT services to migrant women. Given the high prevalence of HIV in Sub-Saharan Africa and high immigration rate in SA as well as the interrelationships that occur between nationals and foreigners such as marriages and other civil unions, infected child of a non-national may transmit the HIV disease to a South African national during adulthood, increasing the health and social cost HIV in SA. Thus, an infant infection averted among non-national is equivalent to averting one potential infection among South African nationals. Therefore, non-provision of care to migrants will impede SA’s fight against HIV/AIDS in the long term. Many studies have established the cost-effectiveness of PMTCT and other HIV services,^30-33^ but in the case of migrants limited evidence exists in South Africa. To our knowledge, this is the first study to estimate the cost-effectiveness, from the South African government’s (payer) perspective, of providing free PMTCT services, through the ANC program, to cross-border migrant women living in SA. While hard-to-reach populations require extra resources to deliver services to them,^34^ the same cannot be said of migrant patients in South Africa since such patients often present themselves for treatment.

## Methods

### Study design

**Figure 1.**
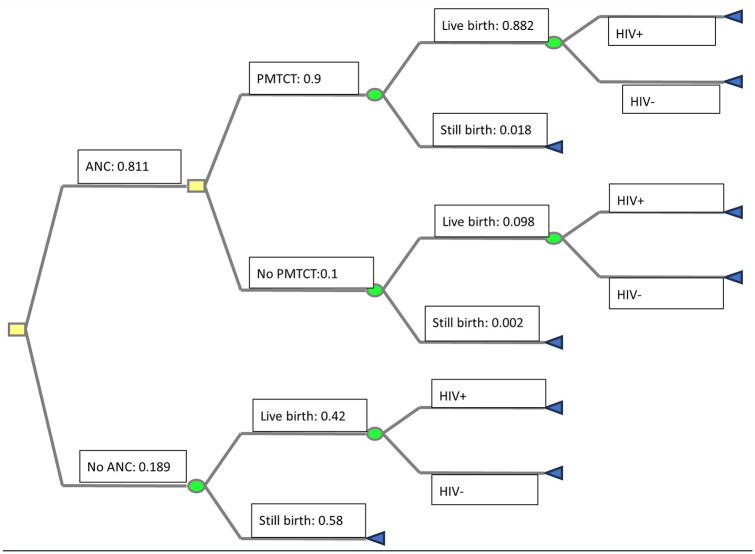
Decision tree for free PMTCT services. Payoffs are calculated using information in Table 1. Abbreviations: ANCm antenatal care; HIV+, HIV positive; HIV−, HIV negative.

A decision tree model (see Figure 1) was designed in Microsoft Excel to simulate the cost-effectiveness of free PMTCT services to migrant mothers in SA through the ANC program. For those who do not attend ANC, postnatal HIV treatment for the mother and the infant will impose additional cost to the health system. The comparator is an ANC program only (without any HIV/PMTCT services). Using available literature (Table 1), we calculated the number of annual HIV positive pregnancies among migrant women. The study is prevalence-based.

**Table 1.** Model data and sources.

Parameter	Base case	Source
Total pregnancies	1 337 632	Moolla et al^35^
Proportion of pregnancies among non-citizens	25%	Moolla et al^35^
Pregnancies (Non-South African citizens)	334 408	Calculation
Proportion of pregnant women who are HIV+ (all nationals)	16.7%	Janse van Rensburg et al^19^, Woldesenbet et al^36^
Number of non-South African pregnancies eligible for the PMTCT	52 762	Calculation
Probabilities		
Antenatal first visit coverage	0.83	Moolla et al^35^
Live birth following ANC	0.98	Moolla et al^35^
Live birth in those without ANC	0.97	Moolla et al^35^
PMTCT Coverage	0.90	Janse van Rensburg et al^19^
Child on lifetime ART	0.90	Assumed
Vertical Transmission rate without PMTCT/similar intervention	0.30	World Health Organisation^5^
Vertical Transmission rate with PMTCT	0.024	Fasawe et al^37^
Outcomes measures		
QALY per infant infection averted	16.88	Zulliger et al^33^
Costs per pregnancy (US$)		
Cost of ANC visit	$11.96	Moolla et al^35^
Treatment cost of infected HIV child on ART per year	$284.10	Meyer-Rath et al^38 a^
PMTCT cost per year	$159.59	Meyer-Rath et al^38 a^

a Costs were adjusted using the US inflation rate.

Abbreviations: ANC, antenatal care; ART, antiretroviral therapy; HIV+, HIV positive; PMTCT, prevention of mother to child transmission; QALY, quality adjusted life years.

### Health outcomes measures

The health outcomes considered in this study are the number of vertical HIV transmissions averted through the PMTCT services and the disability-adjusted life years averted (DALYs) or quality-adjusted life years (QALYs) gained. Following a previous study,^39^ we assumed that a QALY gained is a DALY averted and vice-versa. To obtain the number of vertical transmissions averted, the number of HIV positive pregnant women was multiplied by the difference between the transmission rate without PMTCT intervention and the transmission rate with PMTCT.^40^ We assume that health outcomes for mothers who do not attend ANC will be the same as those who attended ANC but were not enrolled onto the PMTCT program.

### Costs and study perspective

The cost of providing PMTCT services for mothers and the lifetime direct cost for an HIV infected infant were considered. These costs include the cost of all resources needed to provide the service to the mother and if the child is born HIV positive, the cost of resources needed to put the child on ART over a lifetime (ie, up to age 29). We assumed that mothers who do not attend ANC services may use postnatal services. Therefore, infected infants born to mothers who did not attend ANC may be put on the treatment program. All analyses are conducted from a public sector healthcare payer perspective.

### Time horizon

The time horizon for the provision of PMTCT is 1 year. Thus, once the mother starts ANC, PMTCT services will be provided to the pregnant mother for a year. However, an HIV positive child would be expected to be treated over a lifetime. Using the average life expectancy at birth of 29 years, we calculated the lifetime treatment cost of each pediatric infection using the baseline prevalence rates and discounted rate of 5%.

### Sources of data

We sourced per patient cost of ANC visit and lifetime ART cost for infants from previous studies on maternal and child health.^35,37^ The total number of pregnancies annually eligible for free PMTCT services were derived from a previous study,^35^ considering that about 3.1% of all pregnancies in SA are terminated annually.^41^ Table 1 presents the model parameters and their sources of data.

### Cost-effectiveness analysis

We estimated the incremental cost and incremental effectiveness of the ANC_only and ANC + PMTCT interventions. The incremental cost-effectiveness ratio was then calculated. The cost-effectiveness (willingness-to-pay) threshold was determined at US$2081 based on previous studies,^35,42^ and analyses are performed from a public sector payor perspective. All costs are US dollars and may be convert to South African Rand using a conversion rate of ZAR18.5 to the dollar (2024).

### Sensitivity analyses

We performed a one-way sensitivity analysis by varying the inputs. First, the probability of being on PMTCT program given that the migrant has attended the ANC was varied by ±50% of their baseline. Second, we varied the probability or coverage of HIV positive infants who will be on ART for a lifetime to evaluate its effect on the expected cost.

### Budget impact analysis

A budget impact analysis was conducted using information in Table 1 to allow us to capture the financial impact of expanding coverage of PMTCT services to all HIV positive migrant mothers over a period of 5 years. Given that approximately 90% of HIV positive migrant mothers are on PMTCT services, the aim of the budget impact is to estimate the additional spending requirements to service the remaining 10% of the eligible women. For this analysis, we multiply the per patient cost of providing combined ANC and PMTCT services and project this cost over 5-year period (2024-2029). We assume that these migrant population is not hard-to-reach population and therefore the per patient cost of providing care will be the same as that of South African national.

## Results

Based on the literature,^19^ PMTCT coverage is about 90% among HIV positive pregnant women, which is similar to that of South African women. We estimate that without any HIV intervention, vertical transmissions will be 15.83 infections per 1000 live births as against 1.27 infections per 1000 live births under ANC + PMTCT intervention. This means that the ANC + PMTCT intervention will prevent 14.56 pediatric infections in every 1000 live births that occur. This is equivalent to 246 DALYs averted per 1000 live births.

The model predicts that in the absence of no ANC or PMTCT services, the expected cost will be US$73 535 and associated DALYs of 277 in every 1000 live births. The provision of ANC only will be expected to cost US$191 000 and the expected DALYs will be 265. Although the provision of ANC reduces the expected DALYs from 277 to 265, it is not cost-effective if ANC is devoid of HIV treatment such as PMTCT services. Thus, provision of both ANC and PMTCT services to HIV positive migrant women remain cost-effective (Table 2): it produces the lowest DALYs at lower cost thereby producing cost-saving of US$733/DALY averted per 1000 live births.

**Table 2.** Cost-effectiveness results.^a^.

Intervention	Expected cost	Expected DALYs	ICER
No ANC or PMTCT	73 535	277	
ANC Only	190 955	265	9674
ANC + PMTCT	52 889	76	–733

a Per 1000 live births.

Abbreviations: ANC, antenatal care; DALYs, disability adjusted life years; ICER, incremental cost-effectiveness ratio; PMTCT, prevention of mother to child transmission.

The resulting health benefits from the provision of both ANC and PMTCT services were the avoidance of 14 562 new infections among 52 762 HIV positive pregnant women.

### Sensitivity analysis

We performed one-way sensitivity analysis by varying 4 main input variables namely PMTCT coverage rates among pregnant women (scenario 1), the proportion of HIV positive infants that will be put on lifetime ART (scenario 2), the lifetime cost of ART per patient (scenario 3), and ANC cost per woman and the PMTCT cost per woman (scenario 4). Variation in these parameters by ±50% of their baseline values still resulted in the intervention being cost-effective, as incremental cost-effectiveness ratio (ICER) per infection averted was below the US$2081 threshold.^35,42^ Indeed, all scenarios showed that the provision of ANC + PMTCT was cost-saving. The variations in the parameters and the resulting ICER are presented in Appendix Table 1. In terms of the expected cost, raising the PMTCT coverage rate to 100% was associated with about US$17 785 when other parameters are held constant. Similarly, varying the proportion of infected children who will be on lifetime ART to a maximum of 100% resulted in about US$57 529 (see Table 1 in Appendix).

### Financial implications of providing care on the budget

Currently, PMTCT coverage is about 90% among HIV positive migrant mothers. This means that government is spending approx. US$12.4 million on ANC and PMTCT services for migrant women. Given the preventive benefits of PMTCT over the long term, it is imperative for government to increase coverage so that all migrants in need of PMTCT services can access. Covering the excluded 10% of the eligible migrant women population will require additional average spending of US$1.5 million annually over the next 5 years (Table 3).

**Table 3.** Budget impact analysis.

Year	Current coverage		100% coverage		Extra spending
	No. of patients	Cost	No. of patients	Cost	
2024	47 486	12 361 036	52 762	13 734 485	1 373 448
2025	47 989	12 866 825	53 321	14 296 472	1 429 647
2026	48 498	13 393 310	53 887	14 881 456	1 488 146
2027	49 012	13 941 337	54 458	15 490 375	1 549 037
2028	49 531	14 511 789	55 035	16 124 210	1 612 421
2029	50 056	15 105 582	55 618	16 783 981	1 678 398

## Discussion

There are many migrants in SA, some of whom are undocumented and uninsured. Accessing free healthcare services in SA remains a challenge for many foreigners, partly due to poverty and medical xenophobia.^23^ Early access has proven benefits to patients’ health and contributes to a better control of the HIV epidemic by preventing secondary infections. Although previous studies have demonstrated the cost-effectiveness of HIV services for pregnant women,^32,33,43^ there is little or no evidence when it comes to migrants living in SA. This study has analyzed the cost and benefits of providing free PMTCT services to migrant women using a decision tree analysis.

The results show that provision of both ANC and PMTCT services to migrant women remain cost-effective in tackling the HIV epidemic, compared to providing only ANC or no nothing at all. This is because preventing vertical transmission reduces the overall long-term cost associated with perinatal HIV infection, complications and subsequent pediatric HIV care and treatment.^33^ If not prevented, HIV infected children of migrants may transmit HIV once sexually active to both South Africans and non-South Africans and are at risk of opportunistic infections from HIV. This is consistent with findings from France that providing early access to care among migrants living with HIV is cost-effective and can prevent secondary infections.^44^ Aside from the benefits of prevention, the lifetime cost of treating HIV is huge and therefore preventing vertical transmissions will reduce long-term cost associated with HIV treatment and associated opportunistic infections.

Expanding PMTCT coverage with the migrant community will require additional resources. We estimate that overall, an average of US$1.5 million will be needed annually to provide care to the uncovered HIV positive mothers.

### Limitations

This study has limitations. First, we did not consider productivity losses avoided through prevention of vertical transmissions. Most of the parameters used in the model had their data sourced from the literature and therefore may be subject to publication bias. Furthermore, direct non-healthcare costs such as transport associated with receiving care were not considered. Even if services are provided free of charge, such costs might restrict access to usage of ANC and PMTCT services.^32^ Furthermore, our study did not account for the strength of the health system to provide free services. The South African public health system is plagued with many challenges including shortage of medical personnel and inadequate infrastructure, and the high burden of HIV disease which creates an overburdened staff which consequently affects service delivery.^45^ The exclusion of such issues in our analyses may bias the results.

## Conclusion

Approximately 1.33 million pregnancies are recorded in SA annually, with 25% of these pregnancies occurring among non-South African nationals.^35^ At the same time, 30% of all pregnant women receiving ANC in South Africa are HIV positive, posing a challenge for control in a country that is the epicenter of the HIV epidemic. We analyzed the cost-effectiveness of public sector provision of PMTCT and ANC to migrant women using a decision model. Provision of PMTCT services to all in need is a cost-effective measure to prevent vertical transmission and secondary infections. Free provision of maternal and child healthcare is provided to all South Africans. In order for these services not to be overwhelmed, health planning and budgetary allocations must take into account the migrant population as well.

While the provision of care to both nationals and non-nationals living in SA are guaranteed in principle, resources are finite. This demands collective efforts by governments and donor agencies. There is an urgent need to set up a continental healthcare fund to address the healthcare needs of cross-border migrants, especially those in low-income groups, on the continent. This issue has recently come to the attention of SA’s Parliamentary Health Committee. The committee suggests that member countries of the Southern African Development Community (SADC) reimburse SA for healthcare services provided.^46^

While we do not make fixed budget assumptions in this study, we do acknowledge that resources are finite with competing demands on the government which require difficult decisions to be made. This study does demonstrate an economic case to provide PMTCT services to foreign nationals who are HIV positive. Further, although we have not analyzed treatment of other categories of HIV patients, our study suggests that the long-term cost of not treating HIV more generally is likely to be huge in terms of infection control for both migrants and citizens. The government has agreed to treat migrants already in line with legislation and court rulings, and this study demonstrates the economic case that this is an important policy decision that is essential and should be continued. Therefore, measures should be put in place to remove barriers that restrict these women’s access to care.

## Appendix

**Table 1.** One-way sensitivity analysis.

PMTCT coverage (scenario 1)	Model outcomes	0.45	1.00
ICER	–733	– 1,214	–743
Expected cost_ANC + PMTCT	52 889	165 127	17 785
Expected cost_ANC only	190 955	136 542	192 816
HIV+ infants taking ART (scenario 2)	Model outcomes	0.45	1
ICER	–733	– 337	–821
Expected cost_ANC + PMTCT	52 889	32 007	57 529
Expected cost_ANC only	190 955	95 492	212 169
Disc. lifetime cost of infected HIV child on ART (scenario 3)	Model outcomes	2002	6007
ICER	–733	–337	–534.96
Expected cost_ANC + PMTCT	52 889	33 281	43 085
Expected cost_ANC only	190 955	96 798	143 876
ANC cost (scenario 4)	Model outcomes	27.76	83.28
ICER	–733	–734	–732
Expected cost_ANC + PMTCT	52 889	51 431	54 347
Expected cost_ANC only	190 955	189 635	192 275
PMTCT cost (scenario 5)	Model outcomes	102.4	307.19
ICER	–733	–761	–704
Expected cost_ANC + PMTCT	52 889	47 510	58 268
Expected cost_ANC only	190 955	190 955	190 955

Source: authors’.
